# Brain Amyloid Index as a Probable Marker Bridging Between Subjective Memory Complaint and Objective Cognitive Performance

**DOI:** 10.3389/fnins.2022.912891

**Published:** 2022-07-04

**Authors:** Young Min Choe, Guk-Hee Suh, Boung Chul Lee, Ihn-Geun Choi, Jun Ho Lee, Hyun Soo Kim, Jaeuk Hwang, Jee Wook Kim

**Affiliations:** ^1^Department of Neuropsychiatry, Hallym University Dongtan Sacred Heart Hospital, Hwaseong, South Korea; ^2^Department of Psychiatry, Hallym University College of Medicine, Chuncheon, South Korea; ^3^Department of Neuropsychiatry, Hallym University Hangang Sacred Heart Hospital, Seoul, South Korea; ^4^Department of Psychiatry, Seoul W Psychiatric Office, Seoul, South Korea; ^5^Department of Neuropsychiatry, Seoul National University Hospital, Seoul, South Korea; ^6^Department of Laboratory Medicine, Hallym University Dongtan Sacred Heart Hospital, Hwaseong, South Korea; ^7^Department of Psychiatry, Soonchunhyang University Hospital, Seoul, South Korea

**Keywords:** subjective memory complaint, cognition, Alzheimer’s disease, amyloid prediction index, mild cognitive impairment

## Abstract

**Background:**

The association between types of subjective memory complaint (SMC), poor objective cognitive performance, and brain Aβ deposition have been poorly understood. We investigated the association between types of SMC and objective global cognitive performance, then assessed whether this association is mediated by the brain amyloid prediction index (API).

**Methods:**

In total, 173 non-demented older adults [63 cognitively normal (CN) and 110 mild cognitive impairment (MCI)] underwent comprehensive clinical assessments. Objective global cognitive performance and brain amyloid index were measured using the total score (TS) of the Consortium to Establish a Registry for Alzheimer’s Disease neuropsychological battery and API, respectively. In total, four items of SMC from the subjective memory complaints questionnaire (SMCQ) (SMCQ1: a feeling of memory problem; SMCQ2: the feeling of worse memory than 10 years ago; SMCQ3: the feeling of worse memory than others of similar age; or SMCQ4: the feeling of difficulty in everyday life) in global memory function were assessed.

**Results:**

In non-demented and participants with MCI, SMCQ3-positive and SMCQ4-positive groups were associated with decreased TS. In participants with MCI, the SMCQ3-positive group was associated with increased API, and API was associated with decreased TS, but the SMCQ4-positive group did not. In addition, the association between the SMCQ3-positive group and poor TS disappeared when API was controlled as a covariate, indicating that API has a mediation effect.

**Conclusion:**

The present findings suggest that SMC, a feeling of worse memory performance than others in a similar age group, in the older adults without dementia is associated with poor objective cognitive performance *via* increased brain amyloid index.

## Introduction

Subjective memory complaint (SMC) is common in older adults (Jonker et al., 2000; Ramlall et al., 2013), and its prevalence varies from 25 to 50% in community-based studies (O’Connor et al., 1990; Gagnon et al., 1994; Tobiansky et al., 1995; Jonker et al., 1996; Blazer et al., 1997). SMC is associated with several biological and environmental markers including depression (Geerlings et al., 1999; Stewart et al., 2001; Minett et al., 2005; Jessen et al., 2014b), old age (Geerlings et al., 1999; Jessen et al., 2014b), sex (Gillett et al., 2003; Tomita et al., 2014), education (van Oijen et al., 2007; Alagoa Joao et al., 2016), vascular risk (Paradise et al., 2011; Sajjad et al., 2015), and apolipoprotein E (APOE) ε4 (Stewart et al., 2001; Rowe et al., 2010; Jessen et al., 2014b). More importantly, SMC makes older adults worry a lot about Alzheimer’s disease (AD), and there seems to be a clear link between SMC and AD or related cognitive decline.

A growing body of evidence suggests that SMC is a risk factor for AD or related cognitive impairment (Commissaris et al., 1994, 1998; Winblad et al., 2004; Reid and Maclullich, 2006; Reisberg et al., 2008; Jessen et al., 2010, 2014a,2014b; Choe et al., 2018; Janssen et al., 2021; Tort-Merino et al., 2021). In particular, SMC in cognitive normal (CN) state, i.e., subjective cognitive decline (SCD), has been proposed as an early symptom of AD, prior to objective cognitive dysfunction (Winblad et al., 2004; Reid and Maclullich, 2006; Reisberg et al., 2008; Jessen et al., 2010, 2014a,2014b). As brain amyloid-beta (Aβ) deposition is the earliest key pathology of AD (Braak and Braak, 1991; Tobiansky et al., 1995; Jack et al., 2010), SCD may be the first symptomatic manifestation of brain Aβ deposition. Unlike SMC in normal cognition, SMC is an essential component in mild cognitive impairment (MCI), classified as a transitional state between normal aging and dementia (Petersen et al., 1999). As MCI is an obvious risk factor for AD due to the high risk of annual conversion from MCI to AD (Manly et al., 2008; Petersen et al., 2009), MCI has become a critical pre-AD stage for understanding the pathology of AD.

Despite the clinical significance of SMC in preclinical and prodromal AD, its clinical application had been limited in that SMC was not standardized and varied depending on the SMC types. An international working group on the subjective decline in cognition addressed this limitation and presented the characteristics that increase the risk of AD based on the evidence of previous studies (Jessen et al., 2014a). These include a subjective decline in memory, rather than other domains of cognition; concerns (worries) associated with subjective decline (Jessen et al., 2010); and the feeling of worse performance than others of the same age group (Amariglio et al., 2012; Perrotin et al., 2012). However, given the diversity of SMC (Geerlings et al., 1999; Youn et al., 2009; Jessen et al., 2010, 2014a; Amariglio et al., 2012; Perrotin et al., 2012; Choe et al., 2018; Kim et al., 2019), it is not known which types of SMC are associated with AD-related cognitive decline, and whether Aβ deposition mediate the association between SMC and objective cognitive impairment. Taken together, the association between SMC types, poor objective cognitive performance, and brain Aβ deposition have been poorly understood.

Cerebral Aβ deposition is the hallmark pathologic biomarker in AD, and amyloid positron emission tomography (PET) imaging has made it possible to detect cerebral Aβ pathology in living human brains (Klunk et al., 2004). However, due to the high cost and concern about radiation hazards, the application of amyloid PET imaging is still limited in clinical and research settings. Therefore, we developed amyloid prediction index (API) (Lee et al., 2018) derived from commonly available variables including age, sex, education, hypertension, apolipoprotein ε4 (APOE4) positivity, and memory recall score, in memory clinic practice using the data from the Korean Brain Ageing Study for the Early diagnosis and prediction of Alzheimer’s disease (KBASE) cohort (Byun et al., 2017). API showed excellent capability (area under the curve = 0.873) in screening cognitively impaired individuals with cerebral amyloid positivity. API was also externally validated using an independent dataset from the Alzheimer’s Disease Neuroimaging Initiative 2 (ADNI-2) (Petersen et al., 2010), supporting the general applicability of the API.

We first examined the associations of SMC types with objective cognitive performance in non-demented older adults. Regarding SMC types, we selected four items of SMCQ (having a memory problem, memory worsening than 10 years ago, memory worsening than that of others of a similar age, and difficulty in everyday life due to memory decline) designed to assess global memory function from Subjective Memory Complaints Questionnaire (SMCQ) (Youn et al., 2009) because subjective metacognition about global memory function would reflect an early pathological process of AD rather than those items of specific everyday dysfunction. In addition, we examined whether the association of the SMC type(s) with objective cognitive performance is affected by brain amyloid index, i.e., API, which is significantly correlated with SMC type(s) in older adults with MCI.

## Materials and Methods

### Participants

This study was performed as part of the General Lifestyle and AD (GLAD) study, an ongoing prospective cohort study that began in 2020. As of February 2022, 173 individuals (63 adults with CN, and 110 adults with MCI), between 65 and 90 years of age have been enrolled in the GLAD study. Participants were recruited from the community and one recruitment site near Hwaseong, South Korea. Potentially eligible individuals who participated in a dementia screening program at the memory clinic of one university hospital (Hallym University Dongtan Sacred Heart Hospital, Hwaseong, South Korea) were informed about study participation; individuals who volunteered were invited for an assessment of eligibility. In addition, volunteers from the community were recruited through recommendations by other participants, family members, friends, or acquaintances.

For the GLAD study, the cognitive normal group consisted of participants with a Clinical Dementia Rating (Morris, 1993) score of 0 and no diagnosis of MCI or dementia. All participants with MCI met the current consensus criteria for amnestic MCI: memory complaints confirmed by an informant; objective memory impairments; preservation of global cognitive function; independence in functional activities; and absence of dementia. Regarding objective memory impairment, the age-, education-, and sex-adjusted *z*-score was < –1.0 for at least one of four episodic memory tests: Word List Memory, Word List Recall, Word List Recognition, and Constructional Recall tests included in the Korean version of the Consortium to Establish a Registry for Alzheimer’s Disease Assessment Packet (CERAD-K) neuropsychological battery (Morris et al., 1989; Lee et al., 2002, 2004). All participants with MCI had a Clinical Dementia Rating score of 0.5. The exclusion criteria were as follows: presence of a major psychiatric illness and/or significant neurological or medical condition or comorbidity that could affect mental functioning; illiteracy; the presence of significant visual/hearing difficulties and/or severe communication or behavioral problems that would make clinical examinations difficult; and, use of an investigational drug.

This study protocol was approved by the institutional review board of the Hallym University Dongtan Sacred Heart Hospital, it was conducted in accordance with the recommendations of the Declaration of Helsinki. All the participants or their legal representatives provided written informed consent.

#### Clinical Assessments

All the participants underwent standardized clinical assessments by trained psychiatrists based on the GLAD study clinical assessment protocol, which incorporated the Korean version of the CERAD-K (Morris et al., 1989; Lee et al., 2002). A CERAD total score (TS) was generated by summing the scores of six tests in the CERAD neuropsychological battery including the Verbal Fluency, modified Boston Naming Test, Word List Memory, Constructional Praxis, Word List Recall (WLR), and Word List Recognition tests (Seo et al., 2010). The TS was selected as a measure of objective global cognitive function. In addition, TS–WLR was calculated by subtracting the WLR score from TS in order to exclude the effect of WLR.

### Assessment of Subjective Memory Complaint

Four items of SMCs for each participant were assessed based on the answer to the following question on global memory function from the Subjective Memory Complaints Questionnaire (Youn et al., 2009) assessment: SMCQ1: “Do you think that you have a memory problem?”; SMCQ2: “Do you think that your memory is worse than 10 years ago?”; SMCQ3: “Do you think that your memory is worse than that of other people of a similar age?” (Choe et al., 2018); SMCQ4: “Do you feel that your everyday life is difficult due to memory decline?”. The response was recorded as “yes” or “no” to enhance the feasibility and reliability of the item for older adults.

### Measurement of the Amyloid Prediction Index

We measured the API (Lee et al., 2018) for each participant with MCI with commonly available variables, i.e., age, sex, years of education, history of hypertension, APOE4 positivity, and WLR score, in memory clinic practice. The API was defined as the probability for a participant to be amyloid-positive, calculated using the logit value derived from the multivariate logistic regression model for each participant; the details of the calculation on API were described previously (Lee et al., 2018). API derived from this model was proven to be valid across the two cohorts. The area under the curve was 0.873 (95% CI = 0.815–0.918) in the KBASE cohort (Byun et al., 2017), it was 0.808 (95% CI = 0.769–0.842) in the ADNI-2 cohort (Petersen et al., 2010).

### Assessment of Potential Confounders

All participants were evaluated to identify potential confounders, such as depression, vascular risk, body mass index, APOE4 positivity, serum fasting glucose, alcohol intake, and smoking. We used the Geriatric Depression Scale (GDS) (Yesavage et al., 1982; Kim et al., 2008) to measure the severity of depressive symptoms. The comorbidity of vascular risks (e.g., hypertension and diabetes mellitus) was assessed based on data collected by a trained researcher during systematic interviews of participants and their informants. The body mass index was calculated by dividing the weight in kilograms by the square of the height in meters. Lifetime alcohol intake status (never/former/drinker) and smoking status (never/ex-smoker/smoker) were also evaluated through trained researcher interviews and medical record reviews. To acquire accurate information, reliable informants were interviewed. After an overnight fast, blood samples were obtained by venipuncture in the morning (8–9 a.m.). Glucose levels were measured using a COBAS c702 analyzer and dedicated reagents (Roche Diagnostics, Germany). Apolipoprotein E was genotyped using the Seeplex ApoE ACE genotyping kit (Seegene, South Korea). APOE4-positivity was defined as the presence of at least one ε4 allele.

### Statistical Analyses

To examine the associations between SMC and TS, we performed multiple linear regression analyses. For these analyses, we used SMC groups (based on every single SMC item) as the independent variable. The association between SMC and TS may be influenced by various factors. Therefore, we systematically evaluated all the participants to identify potential confounders, such as age, sex, education, depression, APOE4, clinical diagnosis, hypertension, diabetes mellitus, body mass index status, alcohol intake, smoking, and serum fasting glucose. We tested three models to control the covariates in a stepwise manner. The first model included age, sex, education, and GDS status as covariates; the second model included the covariates in the first model plus APOE4, clinical diagnosis, hypertension, and diabetes mellitus; the third model included the covariates in the second model plus body mass index status, alcohol intake, smoking, and serum fasting glucose. We investigated the mediation effects of API on the associations between SMC groups and TS. We first conducted the same regression analyses for the associations between SMC groups and API, then for the associations between SMC groups and TS. We also analyzed the associations between SMC groups and TS using the same regression model while controlling for API as an additional covariate. Referring to the issues of API including objective performance, we performed the same additional analyses in which WLR was used as an additional covariate and in which TS was replaced by TS–WLR in order to exclude the effect of WLR as a component overlaps with TS and API. Referring to the issues of multiple comparisons, the Bonferroni correction method was used for the association of SMC types with TS or API by using a *p*-value < 0.05/the number of SMC types (= 4) as a threshold for statistical significance. *P*-values < 0.05 were considered statistically significant when not otherwise specified. All statistical analyses were performed using IBM SPSS Statistics 27 software (IBM, Armonk, NY, United States).

## Results

### Participant Characteristics

Table 1 presents the demographic and clinical characteristics of the overall participants. Among all participants, 131 (75.72%), 148 (85.55%), 84 (48.55%), and 46 (26.29%) were categorized into the SMCQ1-positive, SMCQ2-positive, SMCQ3-positive, and SMCQ4-positive groups, respectively. Supplementary Tables S1–S3 summarized the demographic and clinical characteristics of overall, participants with MCI and CN according to the SMCQ3 groups, respectively.

**TABLE 1:** Overall participant characteristics.

Characteristic	Overall
*n*	173
Age, y	72.92 (5.62)
Female, *n* (%)	121 (98.37)
**Education, y**	
0–6	62 (36.84)
7–12	80 (46.24)
13–	31(17.92)
MMSE	24.71(3.63)
APOE4 positivity, *n* (%)	36 (20.81)
Clinical diagnosis, MCI, *n* (%)	110 (63.58)
**Geriatric depression scale**	
Normal (<9), *n* (%)	85 (49.13)
Depressed (10), *n* (%)	88 (50.87)
Hypertension, *n* (%)	101 (58.38)
Diabetes mellitus, *n* (%)	39 (22.54)
Body mass index, kg/m^2^	24.71 (3.63)
**Alcohol drink status, *n* (%)**	
Never	97 (56.07)
Former	33 (19.08)
Drinker	42 (24.28)
**Smoking status, *n* (%)**	
Never	131 (75.72)
Former	35 (20.23)
Smoker	6 (3.47)
Glucose, fasting, mg/dL	110.73 (28.57)
**SMCQ positivity, *n* (%)**	
SMCQ1	131 (75.72)
SMCQ2	148 (85.55)
SMCQ3	84 (48.55)
SMCQ4	46 (26.59)
**Objective cognitive performance**	
**Individual score**	
Verbal fluency	12.53 (4.17)
Boston naming test	11.12 (2.65)
Word list memory	13.57 (5.88)
Constructional praxis	9.65 (1.86)
Word list recall (WLR)	4.21 (2.39)
Word list recognition	7.71 (2.48)
**Global score**	
TS score	58.79 (13.98)
TS -WLR score	54.58 (12.19)
Brain amyloid index	
API (*n* = 110)	37.78 (29.26)

*MMSE, mini-mental state examination; APOE4, apolipoprotein ε4; MCI, mild cognitive impairment; Aβ, beta-amyloid; TS, total score of the consortium to establish a registry for Alzheimer’s disease neuropsychological battery; API, amyloid prediction index.*

### Association Between Subjective Memory Complaint and Total Score

**FIGURE 1:**
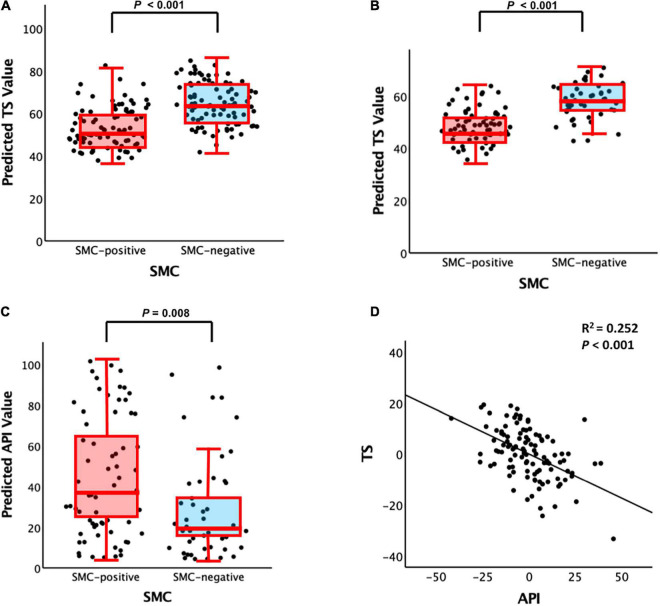
Plots of the associations among SMC (i.e., SMCQ3), API, and TS: **(A)** SMC vs. TS for non-demented participants, **(B)** SMC vs. TS for participants with MCI, **(C)** SMC vs. API for participants with MCI, and **(D)** API vs. TS for participants with MCI. SMC, subjective memory complaint; API, amyloid prediction index; TS, total score of the Consortium to Establish a Registry for Alzheimer’s Disease neuropsychological battery; MCI, mild cognitive impairment. For panels **(A–C)**, box plot controlling for all potential covariates. For panel **(D)**, partial regression plot controlling for all potential covariates are presented.

In non-demented and participants with MCI, SMCQ3- and SMCQ4-positive groups were significantly associated with worse TS compared with those negative groups, respectively (Table 2 and Figures 1A,B). In contrast, no group differences were observed in TS between SMCQ1 and SMCQ2 groups (Table 2).

**TABLE 2:** Results of multiple linear regression analyses for the associations of SMC with objective cognitive performance in CN, MCI, and non-demented older adults.

TS	CN		MCI		CN + MCI	
	β	*p*	β	*p*	β	*p*
**SMCQ1**						
Model 1^a^						
SMC-positive	–0.143	0.207	–0.028	0.748	–0.070	0.319
SMC-negative	Reference		Reference		Reference	
Model 2^b^						
SMC-positive	–0.196	0.088	–0.026	0.772	–0.043	0.448
SMC-negative	Reference		Reference		Reference	
Model 3^c^						
SMC-positive	–0.199	0.102	–0.013	0.885	–0.040	0.472
SMC-negative	Reference		Reference		Reference	
**SMCQ2**						
Model 1^a^						
SMC-positive	0.069	0.548	–0.067	0.448	–0.027	0.698
SMC-negative	Reference		Reference		Reference	
Model 2^b^						
SMC-positive	0.012	0.916	–0.058	0.526	–0.016	0.776
SMC-negative	Reference		Reference		Reference	
Model 3^c^						
SMC-positive	0.031	0.796	–0.037	0.687	0.001	0.979
SMC-negative	Reference		Reference		Reference	
**SMCQ3**						
Model 1^a^						
SMC-positive	–0.150	0.181	–0.350	<0.001*	–0.369	<0.001*
SMC-negative	Reference		Reference		Reference	
Model 2^b^						
SMC-positive	–0.173	0.118	–0.367	<0.001*	–0.240	<0.001*
SMC-negative	Reference		Reference		Reference	
Model 3^c^						
SMC-positive	–0.186	0.110	–0.345	<0.001*	–0.228	<0.001*
SMC-negative	Reference		Reference		Reference	
**SMCQ4**						
Model 1^a^						
SMC-positive	0.152	0.202	–0.305	<0.001*	–0.265	<0.001*
SMC-negative	Reference		Reference		Reference	
Model 2^b^						
SMC-positive	0.149	0.206	–0.299	0.001*	–0.152	0.010*
SMC-negative	Reference		Reference		Reference	
Model 3^c^						
SMC-positive	0.134	0.276	–0.286	0.003*	–0.134	0.024
SMC-negative	Reference		Reference		Reference	

*SMC, subjective memory complaint; CN, cognitive normal; MCI, mild cognitive impairment; CN + MCI, non-demented; TS, total score of the consortium to establish a registry for Alzheimer’s disease neuropsychological battery.*

*^a^Adjusted for age, sex, education, and geriatric depression scale status.*

*^b^Adjusted for covariates in Model 1 plus, apolipoprotein ε4, clinical diagnosis, hypertension, and diabetes mellitus.*

*^c^Adjusted for covariates in Model 2 plus, body mass index status, alcohol intake, smoking, serum fasting glucose.*

*Significant after the Bonferroni correction (i.e., adjusted p < 0.0125 [0.05/4]).

### Association Subjective Memory Complaint, Amyloid Prediction Index, and Total Score

Subjective memory complaints questionnaire 3 positive group alone was significantly associated with increased API in the participants with MCI compared with the SMCQ3-negative group (Table 3 and Figure 1C). In contrast, no group differences were observed in API between the SMCQ1, SMCQ2, and SMCQ4 groups (Table 3). API was significantly associated with decreased TS in the participants with MCI (Table 4 and Figure 1D).

**TABLE 3:** Results of multiple linear regression analyses for the associations of SMC with objective cognitive performance and API in older adults with MCI.

	API	
	β	*p*
**SMCQ1**		
Model 1^a^		
SMC-positive	0.313	0.012*
SMC-negative	Reference	
Model 2^b^		
SMC-positive	0.122	0.143
SMC-negative	Reference	
Model 3^c^		
SMC-positive	0.117	0.161
SMC-negative	Reference	
**SMCQ2**		
Model 1^a^		
SMC-positive	0.241	0.054
SMC-negative	Reference	
Model 2^b^		
SMC-positive	0.098	0.234
SMC-negative	Reference	
Model 3^c^		
SMC-positive	0.146	0.088
SMC-negative	Reference	
**SMCQ3**		
Model 1^a^		
SMC-positive	0.426	<0.001*
SMC-negative	Reference	
Model 2^b^		
SMC-positive	0.233	0.007*
SMC-negative	Reference	
Model 3 ^c^		
SMC-positive	0.231	0.008*
SMC-negative	Reference	
**SMCQ4**		
Model 1^a^		
SMC-positive	0167	0.210
SMC-negative	Reference	
Model 2^b^		
SMC-positive	0.156	0.062
SMC-negative	Reference	
Model 3^c^		
SMC-positive	0.143	0.116
SMC-negative	Reference	

*SMC, subjective memory complaint; Aβ, beta-amyloid; MCI, mild cognitive impairment; TS, total score of the consortium to establish a registry for Alzheimer’s disease neuropsychological battery; API, amyloid prediction index.*

*^a^Adjusted for age, sex, education, and geriatric depression scale status.*

*^b^Adjusted for covariates in Model 1 plus, apolipoprotein ε4, hypertension, and diabetes mellitus.*

*^c^Adjusted for covariates in Model 2 plus, body mass index status, alcohol intake, smoking, serum fasting glucose.*

*Significant after the Bonferroni correction (i.e., adjusted p < 0.0125 [0.05/4]).

**TABLE 4:** Results of multiple linear regression analyses for the associations of API with objective cognitive performance in older adults with MCI.

	TS	
	β	*p*
Model 1^a^		
API	–0.370	0.002
Model 2^b^		
API	–0.810	<0.001
Model 3^c^		
API	–0.842	<0.001

*Aβ, beta-amyloid; MCI, mild cognitive impairment; TS, total score of the consortium to establish a registry for Alzheimer’s disease neuropsychological battery; API, amyloid prediction index.*

*^a^Adjusted for age, sex, education, and geriatric depression scale status.*

*^b^Adjusted for covariates in Model 1 plus, apolipoprotein ε4, hypertension, and diabetes mellitus.*

*^c^Adjusted for covariates in Model 2 plus, body mass index status, alcohol intake, smoking, serum fasting glucose.*

### Effects of Amyloid Prediction Index on the Association Between Subjective Memory Complaint and Total Score

The association between the SMCQ3-positive group and poor TS disappeared in participants with MCI when API was controlled as a covariate, indicating that API has a mediation effect (Table 5). In contrast, there was no mediating effect of API on the association between other SMCQ-positive groups and TS (Table 5).

**TABLE 5:** Results of multiple linear regression analyses for the associations of SMC with objective cognitive performance and API in older adults with MCI.

	TS	
	β	*p*
**SMCQ1**		
Model 1^a^*		
SMC-positive	0.088	0.451
SMC-negative	Reference	
Model 2^b^*		
SMC-positive	0.078	0.477
SMC-negative	Reference	
Model 3^c^*		
SMC-positive	0.083	0.447
SMC-negative	Reference	
**SMCQ2**		
Model 1^a^*		
SMC-positive	–0.040	0.726
SMC-negative	Reference	
Model 2^b^*		
SMC-positive	–0.065	0.539
SMC-negative	Reference	
Model 3^c^*		
SMC-positive	–0.040	0.724
SMC-negative	Reference	
**SMCQ3**		
Model 1^a^*		
SMC-positive	-0.195	0.127
SMC-negative	Reference	
Model 2^b^*		
SMC-positive	–0.200	0.097
SMC-negative	Reference	
Model 3^c^*		
SMC-positive	–0.194	0.109
SMC-negative	Reference	
**SMCQ4**		
Model 1^a^*		
SMC-positive	–0.272	0.018
SMC-negative	Reference	
Model 2^b^*		
SMC-positive	–0.210	0.056
SMC-negative	Reference	
Model 3^c^*		
SMC-positive	–0.200	0.092
SMC-negative	Reference	

*SMC, subjective memory complaint; Aβ, beta-amyloid; MCI, mild cognitive impairment; TS, total score of the consortium to establish a registry for Alzheimer’s disease neuropsychological battery; API, amyloid prediction index.*

*^a^Adjusted for age, sex, education, and geriatric depression scale status.*

*^b^Adjusted for covariates in Model 1 plus, apolipoprotein ε4, hypertension, and diabetes mellitus.*

*^c^Adjusted for covariates in Model 2 plus, body mass index status, alcohol intake, smoking, and serum fasting glucose.*

*Adjusted for covariates in Model plus API.

### Association Among Subjective Memory Complaint, Amyloid Prediction Index, and Total Score With the Independent Effect of Word List Recall

Assuming that the API mediates the association SMCQ3-positive group and TS, it is necessary to exclude the effect of WLR as a component overlaps with TS and API. To demonstrate the independent effect of WLR, we performed the same additional analyses in which WLR was used as an additional covariate and obtained similar results (Supplementary Tables S4–S6). In addition, we performed the same additional analyses in which TS was replaced by TS–WLR and obtained similar results (Supplementary Tables S7–S9).

## Discussion

The result of this study revealed that SMC, defined as a feeling of worse memory performance than others in a similar age group, was significantly associated with a worse objective cognitive performance or brain amyloid index (which are measures of API). In particular, the brain amyloid index had a significant mediation effect on the association between SMC and worse objective cognitive performance. To the best of our knowledge, this is the first study to reveal the mediation effect of brain amyloid index on the association between SMC and objective cognitive dysfunction.

The present study found that SMCQ3 among four SMCQ items is associated with objective global cognitive performance, i.e., TS, *via* increased brain amyloid index, but other SMCQ items did not show the association between subjective complaints, API, and global cognitive decline. As mentioned above, we first assumed that subjective metacognition about global memory function would reflect an early pathological process of AD rather than those items of specific everyday dysfunction, so selected four items of SMCQ were designed to assess global memory function. Among the four SMCQ items, the SMCQ1, “Do you think that you have a memory problem?” did not yield any significant results because of ambiguity and uncertainty, and the SMCQ2, “Do you think that your memory is worse than 10 years ago?”, did not yield any significant results due to the possibility of false positive, being confused with the normal aging process. The SMCQ4, “Do you feel that your everyday life is difficult due to memory decline?”, did not show the association due to its association with impairment of activities of daily living (ADL) in AD rather than MCI. Finally, given less likelihood to confuse ADL impairment or concerns for false positive, we speculated that SMCQ3 would be a more suitable item to provide the information on SMC in the present study. In addition, the international SCD working group supported our finding that SMCQ3 was more informative predictor for objective cognitive performance *via* increased brain amyloid index. They acknowledged the diversity of SMC and suggested AD-related key characteristics of subjective decline (Jessen et al., 2014a), such as subjective memory decline and the feeling of worse performance than others of the same age group (Amariglio et al., 2012; Perrotin et al., 2012), which is similar to the SMCQ3 in the present study.

The finding concerning the association between SMC and objective cognitive performance is consistent with the results of three cohort studies involving non-demented subjects: the Paquid study (Gagnon et al., 1994), the Eastern Baltimore Mental Health Survey (Bassett and Folstein, 1993), and the Berkeley Aging Cohort Study (Vogel et al., 2017). The Paquid cohort (Gagnon et al., 1994) showed that participants with SMC had lower objective cognitive performance than did non-complainers. The Eastern Baltimore Mental Health Survey (Bassett and Folstein, 1993) indicated that 29% of subjective complainers were cognitively impaired, compared with 15% of the non-complainers. The Berkeley Aging Cohort Study (Vogel et al., 2017) revealed that SMC predicted a faster decline in memory performance independently of amyloid-positivity status and interacted with amyloid-positivity status to predict change in memory and global cognition over 4 years. However, the Hughes Hall Project (O’Connor et al., 1990) found no association between SMC and objective performance. These conflicting findings have led to difficulty assessing the association between SMC and objective performance; differences among studies are presumably related to their different types of dementia and distinct definitions of cognitive impairment (O’Connor et al., 1990; Reid and Maclullich, 2006).

Our finding concerning the association between SMC and brain amyloid index is consistent with the results of several studies on SMC–Aβ association using amyloid PET imaging or plasma Aβ measurements in non-demented individuals (Amariglio et al., 2012; Perrotin et al., 2012; Kryscio et al., 2014; Cantero et al., 2016; Kim et al., 2019; Hong et al., 2021; Janssen et al., 2021; Tort-Merino et al., 2021). Although some studies using amyloid PET imaging did not identify a significant SMC–Aβ association (Chetelat et al., 2010; Rodda et al., 2010), these reported a trend toward an SMC–Aβ association (Chetelat et al., 2010; Rodda et al., 2010). These findings suggests that SMC is a risk factor for AD or related cognitive impairment (Commissaris et al., 1994, 1998; Winblad et al., 2004; Reid and Maclullich, 2006; Reisberg et al., 2008; Jessen et al., 2010, 2014a,2014b; Choe et al., 2018; Janssen et al., 2021; Tort-Merino et al., 2021) because Aβ pathology is the earliest key pathology of AD (Braak and Braak, 1991; Tobiansky et al., 1995; Jack et al., 2010). In addition, Aβ may show a mediation effect on the association between SMC and AD or related cognitive impairment.

## Strengths and Limitations

The present study provides the following novel findings. First, we investigated the mediation effect of brain amyloid index and the association between SMC and objective cognitive performance in older adults with MCI. In particular, few studies have investigated their association in older adults with MCI, rather than in CN older adults. Second, to draw a more general conclusion about the association between these factors, we used TS as an indicator of objective cognitive performance, instead of individual cognitive tests that reflect only specific aspects of cognitive performance. Third, we extensively evaluated potential confounders and controlled for them in our statistical models to ensure clarity in our investigations of associations between SMC and objective cognitive performance or brain amyloid index. Nevertheless, this study has some limitations. First, it was a cross-sectional study, which led to difficulty in establishing causal relationships on the base of our findings. To elucidate the nature of the associations among SMC, objective cognitive performance, and brain amyloid deposition, further longitudinal follow-up studies are necessary. Second, we evaluated the API in older adults with MCI using a probability model, instead of brain amyloid PET images. As cognitively intact older adults do not show cognitive impairment and are less likely to have brain amyloid deposition compared with cognitively impaired older adults, it may be difficult to find the association among SMC, API, and cognition. Therefore, this finding is relatively useful for older adults with MCI but not CN. In addition, according to the previous study (Lee et al., 2018), API was defined to be used in cognitively impaired older adults, not in cognitively intact older adults, so it was applied only to MCI older adults in the present study. To exclude the effect of WLR as a component overlaps with TS and API, we performed the same additional analyses in which WLR was used as an additional covariate (Supplementary Tables S3–S5) and in which TS was replaced by TS–WLR (Supplementary Tables S6–S8). These additional analyses showed similar results, respectively, and support that our findings on the association between SMC, API, and TS remain independent of the effect of WLR overlapped with API and TS. Nevertheless, complete elimination of the effects of objective cognitive performance on the association between API and TS was limited because the association could be in part driven by the fact that both include objective cognitive performance. Therefore, the present findings could proceed with the research involving direct assessment of brain Aβ deposition, which has been difficult to perform due to high cost or concern about radiation hazards and will clarify the mechanisms underlying the association between SMC and objective cognitive performance. At last, there was a possibility of a crossover between the SMC types. In the broad framework of SMC, cross-over between each SMC type may be bound to be high. This possibility may call into question the classification approach. It may be necessary to classify the SMC types by fully considering these issues in future studies.

## Conclusion

The present findings suggest that SMC, a feeling of worse memory performance than others in a similar age group, in older adults without dementia is associated with poor objective cognitive performance *via* increased brain amyloid index.

## Data Availability Statement

The study data are not freely accessible because the IRB of the Hallym University Dongtan Sacred Heart Hospital prevents public sharing of such data for privacy reasons. However, the data are available on reasonable request after IRB approval. Requests for data access can be submitted to an independent administrative coordinator by e-mail yoon4645@gmail.com.

## Ethics Statement

The studies involving human participants were reviewed and approved by Hallym University Dongtan Sacred Heart Hospital. The patients/participants provided their written informed consent to participate in this study.

## Author Contributions

JK conceived and designed the study. YC, G-HS, BL, I-GC, JL, HK, JH, and JK were involved in the acquisition, or analysis, and interpretation of the data and helped to draft the manuscript. JK, YC, G-HS, BL, I-GC, JL, HK, and JH were major contributors to writing the manuscript and critically revising the manuscript for intellectual content. JK served as principal investigator and supervised the study. All authors read and approved the final manuscript.

## Conflict of Interest

The authors declare that the research was conducted in the absence of any commercial or financial relationships that could be construed as a potential conflict of interest.

## Publisher’s Note

All claims expressed in this article are solely those of the authors and do not necessarily represent those of their affiliated organizations, or those of the publisher, the editors and the reviewers. Any product that may be evaluated in this article, or claim that may be made by its manufacturer, is not guaranteed or endorsed by the publisher.
