# Supplementary material for: Brain Amyloid Index as a Probable Marker Bridging Between Subjective Memory Complaint and Objective Cognitive Performance
Source: Front Neurosci. 2022 Jul 4;16:912891. doi: 10.3389/fnins.2022.912891 (PMC9289513; doi:10.3389/fnins.2022.912891)
Supplement: Supplementary file 1 [file Data_Sheet_1.docx]

**SUPPLEMENTARY MATERIALS**

**Table S1.** Overall participant characteristics according to SMCQ3

**Table S2.** MCI participant characteristics according to SMCQ3

**Table S3.** CN participant characteristics according to SMCQ3

**Table S4.** Results of multiple linear regression analyses for the associations of SMC with objective cognitive performance in MCI older adults under adjustment for WLR

**Table S5.** Results of multiple linear regression analyses for the associations of API with objective cognitive performance in MCI older adults under adjustment for WLR

**Table S6.** Results of multiple linear regression analyses for the associations of SMC with objective cognitive performance and API in MCI older adults under adjustment for WLR

**Table S7.** Results of multiple linear regression analyses for the associations of SMC with objective cognitive performance (TS-WLR) and API in CN, MCI, and non-demented older adults

**Table S8.** Results of multiple linear regression analyses for the associations of API with objective cognitive performance (TS-WLR) in MCI older adults

**Table S9.** Results of multiple linear regression analyses for the associations of SMC with objective cognitive performance (TS-WLR) and API in MCI older adults

This supplementary material has been provided by the authors to give readers additional information about their work.

| **Table S1.** Overall participant characteristics according to SMCQ3 | | | | | |  |
| --- | --- | --- | --- | --- | --- | --- |
| Characteristic | | Subjective memory complaint (SMCQ3) | | | *t* or *χ^2^* | *p* |
|  |  | Positive | Negative | Total |  |  |
| n | | 84 | 89 | 173 |  |  |
| Age, y | | 73.42 (5.78) | 72.46 (5.45) | 72.92 (5.62) | 1.120 | 0.264 ^a^ |
| Female, n (%) | | 54 (64.29) | 67 (75.28) | 121 (98.37) | 2.485 | 0.115 ^b^ |
| Education, y | |  |  |  | 3.852 | 0.146 ^b^ |
| 0-6 | | 28 (33.33) | 34 (38.20) | 62 (36.84) |  |  |
| 7-12 | | 36 (42.86) | 44 (49.44) | 80 (46.24) |  |  |
| 13- | | 20 (23.81) | 11 (12.36) | 31 (17.92) |  |  |
| MMSE | | 23.55 (3.35) | 25.80 (3.55) | 24.71 (3.63) | -4.279 | <0.001 ^a^ |
| APOE4 positivity, n (%) | | 22 (26.19) | 14 (15.73) | 36 (20.81) | 2.745 | 0.098 ^b^ |
| Clinical diagnosis, MCI, n (%) | | 65 (77.38) | 45 (50.56) | 110 (63.58) | 13.424 | <0.001 ^b^ |
| Geriatric depression scale | |  |  |  | 3.642 | 0.056 ^b^ |
| Normal (<9), n (%) | | 35 (41.67) | 50 (56.18) | 85 (49.13) |  |  |
| Depressed ($\geq$10), n (%) | | 49 (58.33) | 39 (43.82) | 88 (50.87) |  |  |
| Hypertension, n (%) | | 47 (55.95) | 54 (60.67) | 101 (58.38) | 0.397 | 0.529 ^b^ |
| Diabetes mellitus, n (%) | | 17 (20.24) | 22 (24.72) | 39 (22.54) | 0.497 | 0.481 ^b^ |
| Body mass index, kg/m^2^ | | 25.21 (3.39) | 24.50 (3.92) | 24.71 (3.63) | 1.275 | 0.204 ^a^ |
| Alcohol drink status, n (%) | |  |  |  | 0.559 | 0.756 ^b^ |
| Never | | 48 (57.14) | 49 (55.06) | 97 (56.07) |  |  |
| Former | | 14 (16.67) | 19 (21.35) | 33 (19.08) |  |  |
| Drinker | | 21 (25.00) | 21 (23.60) | 42 (24.28) |  |  |
| Smoking status, n (%) | |  |  |  | 1.636 | 0.454 ^c^ |
| Never | | 60 (71.43) | 71 (79.78) | 131 (75.72) |  |  |
| Former | | 19 (22.62) | 16 (17.98) | 35 (20.23) |  |  |
| Smoker | | 4 (4.76) | 2 (2.25) | 6 (3.47) |  |  |
| Glucose, fasting, mg/dL | | 108.80 (32.61) | 112.58 (24.14) | 110.73 (28.57) | -0.867 | 0.387 ^a^ |
| Objective cognitive performance | |  |  |  |  |  |
| Individual score | |  |  |  |  |  |
| Verbal fluency | | 11.85 (4.17) | 13.17 (4.09) | 12.53 (4.17) | -2.106 | 0.037 ^a^ |
| Boston naming test | | 10.67 (2.64) | 11.55 (2.61) | 11.12 (2.65) | -2.217 | 0.028 ^a^ |
| Word list memory | | 11.27 (6.70) | 15.73 (3.94) | 13.57 (5.88) | -5.290 | <0.001 ^a^ |
| Constructional praxis | | 9.42 (1.78) | 9.87 (1.91) | 9.65 (1.86) | -1.594 | 0.113 ^a^ |
| Word list recall (WLR) | | 3.46 (2.37) | 4.91 (2.20) | 4.21 (2.39) | -4.161 | <0.001 ^a^ |
| Word list recognition | | 6.68 (2.82) | 8.69 (1.59) | 7.71 (2.48) | -5.717 | <0.001 ^a^ |
| Global score | |  |  |  |  |  |
| TS score | | 53.37 (13.83) | 63.91 (12.12) | 58.79 (13.98) | -5.340 | <0.001 ^a^ |
| TS -WLR score | | 49.90 (12.23) | 59.00 (10.43) | 54.58 (12.19) | -5.272 | <0.001 ^a^ |
| Brain amyloid index | |  |  |  |  |  |
| API (n = 110) | | 44.28 (29.92) | 28.40 (25.80) | 37.78 (29.26) | 2.891 | 0.005 ^a^ |
| MMSE = mini-mental state examination, APOE4 = apolipoprotein ε4, MCI = mild cognitive impairment. Aβ=beta-amyloid, TS = total score of the consortium to establish a registry for Alzheimer’s disease neuropsychological battery, API = amyloid prediction index.  ^a^ by t-test.  ^b^ by chi-square test.  ^c^ by fisher exact test. | | | | | | |

| **Table S2.** MCI participant characteristics according to SMCQ3 | | | | | |  |
| --- | --- | --- | --- | --- | --- | --- |
| Characteristic | | Subjective memory complaint (SMCQ3) | | | *t* or *χ^2^* | *p* |
|  |  | Positive | Negative | Total |  |  |
| n | | 65 | 45 | 110 |  |  |
| Age, y | | 73.66 (5.78) | 72.53 (5.71) | 73.20 (5.75) | 1.012 | 0.314 ^a^ |
| Female, n (%) | | 44 (67.69) | 33 (73.33) | 77 (70.00) |  | 0.526 ^b^ |
| Education, y | |  |  |  | 2.943 | 0.230 ^b^ |
| 0-6 | | 23 (35.38) | 21 (46.67) | 44 (40.00) |  |  |
| 7-12 | | 27 (41.54) | 19 (42.22) | 46 (41.82) |  |  |
| 13- | | 15 (23.08) | 5 (11.11) | 20 (18.18) |  |  |
| MMSE | | 22.71 (3.05) | 24.38 (4.08) | 23.39 (3.58) | -2.458 | 0.016 ^a^ |
| APOE4 positivity, n (%) | | 20 (30.77) | 8 (17.78) | 28 (25.45) | 2.365 | 0.124 ^b^ |
| Clinical diagnosis, MCI, n (%) | | 65 (100.00) | 45 (100.00) | 110 (100.00) |  |  |
| Geriatric depression scale | |  |  |  | 2.703 | 0.100 ^b^ |
| Normal (<9), n (%) | | 23 (35.38) | 23 (51.11) | 46 (41.82) |  |  |
| Depressed ($\geq$10), n (%) | | 42 (64.62) | 22 (48.89) | 64 (58.18) |  |  |
| Hypertension, n (%) | | 37 (56.92) | 28 (62.22) | 65 (59.09) | 0.309 | 0.578 ^b^ |
| Diabetes mellitus, n (%) | | 13 (20.00) | 14 (31.11) | 27 (24.55) | 1.773 | 0.183 ^b^ |
| Body mass index, kg/m^2^ | | 25.36 (3.57) | 24.15 (4.89) | 24.86 (4.19) | 1.494 | 0.138 ^a^ |
| Alcohol drink status, n (%) | |  |  |  | 1.290 | 0.525 ^b^ |
| Never | | 36 (52.17) | 21 (46.67) | 57 (51.82) |  |  |
| Former | | 13 (20.00) | 13 (28.89) | 26 (23.64) |  |  |
| Drinker | | 15 (23.08) | 11 (24.44) | 26 (23.64) |  |  |
| Smoking status, n (%) | |  |  |  | 0.498 | 0.812 ^c^ |
| Never | | 47 (72.31) | 33 (73.33) | 80 (72.72) |  |  |
| Former | | 14 (21.54) | 11 (24.44) | 25 (22.73) |  |  |
| Smoker | | 3 (4.62) | 1 (2.22) | 4 (3.64) |  |  |
| Glucose, fasting, mg/dL | | 110.95 (34.30) | 112.71 (23.64) | 111.67 (30.29) | -0.298 | 0.766 ^a^ |
| Objective cognitive performance | |  |  |  |  |  |
| Individual score | |  |  |  |  |  |
| Verbal fluency | | 11.08 (3.77) | 11.31 (3.42) | 11.17 (3.62) | -0.333 | 0.740 ^a^ |
| Boston naming test | | 10.15 (2.64) | 10.69 (3.04) | 10.37 (2.81) | -0.981 | 0.329 ^a^ |
| Word list memory | | 9.42 (6.19) | 13.78 (3.81) | 11.20 (5.74) | -4.571 | <0.001 ^a^ |
| Constructional praxis | | 9.23 (1.86) | 9.44 (2.37) | 9.32 (2.08) | -0.529 | 0.598 ^a^ |
| Word list recall (WLR) | | 2.74 (2.00) | 3.82 (2.22) | 3.18 (2.15) | -2.670 | 0.009 ^a^ |
| Word list recognition | | 6.08 (2.88) | 8.13 (1.95) | 6.92 (2.73) | -4.461 | <0.001 ^a^ |
| Global score | |  |  |  |  |  |
| TS score | | 48.74 (11.13) | 57.17 (11.23) | 52.19 (11.88) | -3.895 | <0.001 ^a^ |
| TS -WLR score | | 46.00 (10.17) | 53.36 (9.77) | 49.01 (9.77) | -3.790 | <0.001 ^a^ |
| Brain amyloid index | |  |  |  |  |  |
| API | | 44.28 (29.92) | 28.40 (25.80) | 37.78 (29.26) | 2.891 | 0.005 ^a^ |
| MMSE = mini-mental state examination, APOE4 = apolipoprotein ε4, MCI = mild cognitive impairment. Aβ=beta-amyloid, TS = total score of the consortium to establish a registry for Alzheimer’s disease neuropsychological battery, API = amyloid prediction index.  ^a^ by t-test.  ^b^ by chi-square test.  ^c^ by fisher exact test. | | | | | | |

| **Table S3.** CN participant characteristics according to SMCQ3 | | | | | |  |
| --- | --- | --- | --- | --- | --- | --- |
| Characteristic | | Subjective memory complaint (SMCQ3) | | | *t* or *χ^2^* | *p* |
|  |  | Positive | Negative | Total |  |  |
| n | | 19 | 44 | 63 |  |  |
| Age, y | | 72.58 (5.88) | 72.39 (5.23) | 72.44 (5.39) | 0.129 | 0.898 ^a^ |
| Female, n (%) | | 10 (52.63) | 34 (77.27) | 44 (69.84) | 3.825 | 0.050 ^b^ |
| Education, y | |  |  |  | 1.490 | 0.475 ^b^ |
| 0-6 | | 5 (26.32) | 13 (29.55) | 18 (28.57) |  |  |
| 7-12 | | 9 (47.37) | 25 (56.82) | 34 (53.97) |  |  |
| 13- | | 5 (26.32) | 6 (13.64) | 11 (17.46) |  |  |
| MMSE | | 26.42 (2.76) | 27.25 (2.13) | 27.00 (2.34) | -1.297 | 0.200 ^a^ |
| APOE4 positivity, n (%) | | 2 (10.53) | 6 (13.64) | 8 (12.70) | 0.138 | 1.000 ^c^ |
| Clinical diagnosis, MCI, n (%) | | 0 (0.00) | 0 (0.00) | 0 (0.00) |  |  |
| Geriatric depression scale | |  |  |  | 0.018 | 0.893 ^b^ |
| Normal (<9), n (%) | | 12 (63.16) | 27 (61.36) | 39 (61.90) |  |  |
| Depressed ($\geq$10), n (%) | | 7 (36.84) | 17 (38.64) | 24 (38.10) |  |  |
| Hypertension, n (%) | | 10 (52.63) | 26 (59.09) | 36 (57.14) | 0.226 | 0.634 ^b^ |
| Diabetes mellitus, n (%) | | 4 (21.05) | 8 (18.18) | 12 (19.05) | 0.071 | 1.000 ^c^ |
| Body mass index, kg/m^2^ | | 24.72 (2.68) | 24.86 (2.58) | 24.82 (2.59) | -0.192 | 0.848 ^a^ |
| Alcohol drink status, n (%) | |  |  |  | 1.124 | 0.602 ^c^ |
| Never | | 12 (63.16) | 28 (63.64) | 40 (63.49) |  |  |
| Former | | 1 (5.26) | 6 (13.64) | 7 (11.11) |  |  |
| Drinker | | 6 (3.16) | 10 (22.73) | 16 (25.40) |  |  |
| Smoking status, n (%) | |  |  |  | 3.136 | 0.176 ^c^ |
| Never | | 13 (68.42) | 38 (86.36) | 51 (80.95) |  |  |
| Former | | 5 (26.32) | 5 (11.36) | 10 (15.87) |  |  |
| Smoker | | 1 (5.26) | 1 (2.27) | 2 (3.17) |  |  |
| Glucose, fasting, mg/dL | | 101.42 (25.39) | 112.44 (24.92) | 109.06 (3.86) | -1.596 | 0.116 ^a^ |
| Objective cognitive performance | |  |  |  |  |  |
| Individual score | |  |  |  |  |  |
| Verbal fluency | | 14.47 (4.50) | 15.07 (3.87) | 14.89 (4.04) | -0.532 | 0.596 ^a^ |
| Boston naming test | | 12.42 (1.74) | 12.43 (1.69) | 12.43 (1.69) | -0.023 | 0.982 ^a^ |
| Word list memory | | 17.63 (4.00) | 17.73 (2.98) | 17.70 (3.29) | -0.105 | 0.917 ^a^ |
| Constructional praxis | | 10.05 (1.31) | 10.30 (1.17) | 10.22 (1.21) | -0.728 | 0.470 ^a^ |
| Word list recall (WLR) | | 5.95 (1.78) | 6.02 (1.55) | 6.00 (1.61) | -0.170 | 0.866 ^a^ |
| Word list recognition | | 8.74 (1.15) | 9.25 (0.81) | 9.10 (0.95) | -1.769 | 0.047 ^a^ |
| Global score | |  |  |  |  |  |
| TS score | | 69.21 (9.95) | 70.80 (8.68) | 70.32 (9.03) | -0.636 | 0.527 ^a^ |
| TS -WLR score | | 63.26 (8.86) | 64.77 (7.60) | 64.32 (7.96) | -0.688 | 0.494 ^a^ |
| MMSE = mini-mental state examination, APOE4 = apolipoprotein ε4, MCI = mild cognitive impairment. Aβ=beta-amyloid, TS = total score of the consortium to establish a registry for Alzheimer’s disease neuropsychological battery, API = amyloid prediction index.  ^a^ by t-test.  ^b^ by chi-square test.  ^c^ by fisher exact test. | | | | | | |

| **Table S4.** Results of multiple linear regression analyses for the associations of SMC with objective cognitive performance and API in MCI older adults under adjustment for WLR | | | |
| --- | --- | --- | --- |
|  | API | |  |
|  | $\beta$ | *p* |  |
| **SMCQ1** |  |  |  |
| Model 1 ^a, *^ |  |  |  |
| SMC-positive | 0.268 | 0.010 |  |
| SMC-negative | Reference |  |  |
| Model 2 ^b, *^ |  |  |  |
| SMC-positive | 0.103 | 0.127 |  |
| SMC-negative | Reference |  |  |
| Model 3 ^c, *^ |  |  |  |
| SMC-positive | 0.104 | 0.137 |  |
| SMC-negative | Reference |  |  |
| **SMCQ2** |  |  |  |
| Model 1 ^a, *^ |  |  |  |
| SMC-positive | 0.189 | 0.022 |  |
| SMC-negative | Reference |  |  |
| Model 2 ^b, *^ |  |  |  |
| SMC-positive | 0.057 | 0.284 |  |
| SMC-negative | Reference |  |  |
| Model 3 ^c, *^ |  |  |  |
| SMC-positive | 0.061 | 0.259 |  |
| SMC-negative | Reference |  |  |
| **SMCQ3** |  |  |  |
| Model 1 ^a, *^ |  |  |  |
| SMC-positive | 0.236 | 0.005 |  |
| SMC-negative | Reference |  |  |
| Model 2 ^b, *^ |  |  |  |
| SMC-positive | 0.111 | 0.039 |  |
| SMC-negative | Reference |  |  |
| Model 3 ^c, *^ |  |  |  |
| SMC-positive | 0.116 | 0.036 |  |
| SMC-negative | Reference |  |  |
| **SMCQ4** |  |  |  |
| Model 1 ^a, *^ |  |  |  |
| SMC-positive | 0.056 | 0.535 |  |
| SMC-negative | Reference |  |  |
| Model 2 ^b, *^ |  |  |  |
| SMC-positive | 0.013 | 0.820 |  |
| SMC-negative | Reference |  |  |
| Model 3 ^c, *^ |  |  |  |
| SMC-positive | 0.016 | 0.786 |  |
| SMC-negative | Reference |  |  |

SMC=subjective memory complaint, Aβ=beta-amyloid, MCI=mild cognitive impairment, TS = total score of the consortium to establish a registry for Alzheimer’s disease neuropsychological battery, API = amyloid prediction index, WLR=word list recall.

^a^ Adjusted for age, sex, education, and geriatric depression scale status.

^b^ Adjusted for covariates in Model 1 plus, apolipoprotein ε4, hypertension, and diabetes mellitus.

^c^ Adjusted for covariates in Model 2 plus, body mass index status, alcohol intake, smoking, serum fasting glucose.

^*^Adjusted for covariates in Model plus WLR.

| **Table S5.** Results of multiple linear regression analyses for the associations of API with objective cognitive performance in MCI older adults under adjustment for WLR | | | |
| --- | --- | --- | --- |
|  | TS | |  |
|  | $\beta$ | *p* |  |
| Model 1 ^a, *^ |  |  |  |
| API | -0.203 | 0.015 |  |
| Model 2 ^b, *^ |  |  |  |
| API | -0.467 | 0.008 |  |
| Model 3 ^c, *^ |  |  |  |
| API | -0.452 | 0.010 |  |

Aβ=beta-amyloid, MCI=mild cognitive impairment, TS=total score of the consortium to establish a registry for Alzheimer’s disease neuropsychological battery, API = amyloid prediction index.

^a^ Adjusted for age, sex, education, and geriatric depression scale status.

^b^ Adjusted for covariates in Model 1 plus, apolipoprotein ε4, hypertension, and diabetes mellitus.

^c^ Adjusted for covariates in Model 2 plus, body mass index status, alcohol intake, smoking, serum fasting glucose.

^*^Adjusted for covariates in Model plus WLR.

| **Table S6.** Results of multiple linear regression analyses for the associations of SMC with objective cognitive performance and API in MCI older adults under adjustment for WLR | | | |
| --- | --- | --- | --- |
|  | TS | |  |
|  | $\beta$ | *p* |  |
| **SMCQ1** |  |  |  |
| Model 1 ^a, *^ |  |  |  |
| SMC-positive | 0.015 | 0.865 |  |
| SMC-negative | Reference |  |  |
| Model 2 ^b, *^ |  |  |  |
| SMC-positive | 0.027 | 0.769 |  |
| SMC-negative | Reference |  |  |
| Model 3 ^c, *^ |  |  |  |
| SMC-positive | 0.033 | 0.718 |  |
| SMC-negative | Reference |  |  |
| **SMCQ2** |  |  |  |
| Model 1 ^a, *^ |  |  |  |
| SMC-positive | -0.001 | 0.989 |  |
| SMC-negative | Reference |  |  |
| Model 2 ^b, *^ |  |  |  |
| SMC-positive | -0.019 | 0.832 |  |
| SMC-negative | Reference |  |  |
| Model 3 ^c, *^ |  |  |  |
| SMC-positive | -0.007 | 0.943 |  |
| SMC-negative | Reference |  |  |
| **SMCQ3** |  |  |  |
| Model 1 ^a, *^ |  |  |  |
| SMC-positive | -0.133 | 0.177 |  |
| SMC-negative | Reference |  |  |
| Model 2 ^b, *^ |  |  |  |
| SMC-positive | -0.150 | 0.137 |  |
| SMC-negative | Reference |  |  |
| Model 3 ^c, *^ |  |  |  |
| SMC-positive | -0.150 | 0.143 |  |
| SMC-negative | Reference |  |  |
| **SMCQ4** |  |  |  |
| Model 1 ^a, *^ |  |  |  |
| SMC-positive | -0.131 | 0.160 |  |
| SMC-negative | Reference |  |  |
| Model 2 ^b, *^ |  |  |  |
| SMC-positive | -0.131 | 0.160 |  |
| SMC-negative | Reference |  |  |
| Model 3 ^c, *^ |  |  |  |
| SMC-positive | -0.139 | 0.176 |  |
| SMC-negative | Reference |  |  |

SMC=subjective memory complaint, Aβ=beta-amyloid, MCI=mild cognitive impairment, TS = total score of the consortium to establish a registry for Alzheimer’s disease neuropsychological battery, API = amyloid prediction index, WLR=word list recall.

^a^ Adjusted for age, sex, education, and geriatric depression scale status.

^b^ Adjusted for covariates in Model 1 plus, apolipoprotein ε4, hypertension, and diabetes mellitus.

^c^ Adjusted for covariates in Model 2 plus, body mass index status, alcohol intake, smoking, serum fasting glucose.

^*^Adjusted for covariates in Model plus API and WLR.

| **Table S7.** Results of multiple linear regression analyses for the associations of SMC with objective cognitive performance (TS-WLR) in CN, MCI, and non-demented older adults | | | | | | | |
| --- | --- | --- | --- | --- | --- | --- | --- |
| TS-WLR | CN | | MCI | | CN+MCI | |  |
|  | $\beta$ | *p* | $\beta$ | *p* | $\beta$ | *p* |  |
| **SMCQ1** |  |  |  |  |  |  |  |
| Model 1 ^a^ |  |  |  |  |  |  |  |
| SMC-positive | -0.170 | 0.133 | -0.023 | 0.793 | -0.070 | 0.317 |  |
| SMC-negative | Reference |  | Reference |  | Reference |  |  |
| Model 2 ^b^ |  |  |  |  |  |  |  |
| SMC-positive | -0.225 | 0.050 | -0.021 | 0.819 | -0.043 | 0.450 |  |
| SMC-negative | Reference |  | Reference |  | Reference |  |  |
| Model 3 ^c^ |  |  |  |  |  |  |  |
| SMC-positive | -0.223 | 0.068 | -0.009 | 0.922 | -0.041 | 0.470 |  |
| SMC-negative | Reference |  | Reference |  | Reference |  |  |
| **SMCQ2** |  |  |  |  |  |  |  |
| Model 1 ^a^ |  |  |  |  |  |  |  |
| SMC-positive | 0.060 | 0.600 | -0.048 | 0.586 | -0.017 | 0.810 |  |
| SMC-negative | Reference |  | Reference |  | Reference |  |  |
| Model 2 ^b^ |  |  |  |  |  |  |  |
| SMC-positive | 0.004 | 0.974 | -0.044 | 0.634 | -0.006 | 0.910 |  |
| SMC-negative | Reference |  | Reference |  | Reference |  |  |
| Model 3 ^c^ |  |  |  |  |  |  |  |
| SMC-positive | 0.026 | 0.831 | -0.021 | 0.817 |  |  |  |
| SMC-negative | Reference |  | Reference |  | Reference | 0.841 |  |
| **SMCQ3** |  |  |  |  |  |  |  |
| Model 1 ^a^ |  |  |  |  |  |  |  |
| SMC-positive | -0.160 | 0.153 | -0.341 | <0.001 | -0.366 | <0.001 |  |
| SMC-negative | Reference |  | Reference |  | Reference |  |  |
| Model 2 ^b^ |  |  |  |  |  |  |  |
| SMC-positive | -0.182 | 0.100 | -0.361 | <0.001 | -0.244 | <0.001 |  |
| SMC-negative | Reference |  | Reference |  | Reference |  |  |
| Model 3 ^c^ |  |  |  |  |  |  |  |
| SMC-positive | -0.191 | 0.102 | -0.335 | <0.001 | -0.230 | <0.001 |  |
| SMC-negative | Reference |  | Reference |  | Reference |  |  |
| **SMCQ4** |  |  |  |  |  |  |  |
| Model 1 ^a^ |  |  |  |  |  |  |  |
| SMC-positive | 0.166 | 0.163 | -0.285 | 0.001 | -0.252 | <0.001 |  |
| SMC-negative | Reference |  | Reference |  | Reference |  |  |
| Model 2 ^b^ |  |  |  |  |  |  |  |
| SMC-positive | 0.163 | 0.166 | -0.288 | 0.002 | -0.146 | 0.016 |  |
| SMC-negative | Reference |  | Reference |  | Reference |  |  |
| Model 3 ^c^ |  |  |  |  |  |  |  |
| SMC-positive | 0.157 | 0.202 | -0.275 | 0.004 | -0.128 | 0.035 |  |
| SMC-negative | Reference |  | Reference |  | Reference |  |  |

SMC=subjective memory complaint, CN=cognitive normal, MCI=mild cognitive impairment, CN+MCI=non-demented, TS = total score of the consortium to establish a registry for Alzheimer’s disease neuropsychological battery, WLR= word list recall.

^a^ Adjusted for age, sex, education, and geriatric depression scale status.

^b^ Adjusted for covariates in Model 1 plus, apolipoprotein ε4, clinical diagnosis, hypertension, and diabetes mellitus.

^c^ Adjusted for covariates in Model 2 plus, body mass index status, alcohol intake, smoking, serum fasting glucose.

| **Table S8.** Results of multiple linear regression analyses for the associations of API with objective cognitive performance (TS-WLR) in MCI older adults | | | |
| --- | --- | --- | --- |
|  | TS-WLR | |  |
|  | $\beta$ | *p* |  |
| Model 1 ^a^ |  |  |  |
| API | -0.297 | 0.015 |  |
| Model 2 ^b^ |  |  |  |
| API | -0.685 | <0.001 |  |
| Model 3 ^c^ |  |  |  |
| API | -0.713 | <0.001 |  |

Aβ=beta-amyloid, MCI=mild cognitive impairment, TS = total score of the consortium to establish a registry for Alzheimer’s disease neuropsychological battery, WLR= word list recall, API = amyloid prediction index.

^a^ Adjusted for age, sex, education, and geriatric depression scale status.

^b^ Adjusted for covariates in Model 1 plus, apolipoprotein ε4, hypertension, and diabetes mellitus.

^c^ Adjusted for covariates in Model 2 plus, body mass index status, alcohol intake, smoking, serum fasting glucose.

| **Table S9.** Results of multiple linear regression analyses for the associations of SMC with objective cognitive performance (TS-WLR) and API in MCI older adults | | | |
| --- | --- | --- | --- |
|  | TS - WLR | |  |
|  | $\beta$ | *p* |  |
| **SMCQ1** |  |  |  |
| Model 1 ^a, *^ |  |  |  |
| SMC-positive | 0.079 | 0.512 |  |
| SMC-negative | Reference |  |  |
| Model 2 ^b, *^ |  |  |  |
| SMC-positive | 0.073 | 0.525 |  |
| SMC-negative | Reference |  |  |
| Model 3 ^c, *^ |  |  |  |
| SMC-positive | 0.078 | 0.493 |  |
| SMC-negative | Reference |  |  |
| **SMCQ2** |  |  |  |
| Model 1 ^a, *^ |  |  |  |
| SMC-positive | -0.034 | 0.771 |  |
| SMC-negative | Reference |  |  |
| Model 2 ^b, *^ |  |  |  |
| SMC-positive | -0.060 | 0.590 |  |
| SMC-negative | Reference |  |  |
| Model 3 ^c, *^ |  |  |  |
| SMC-positive | -0.035 | 0.769 |  |
| SMC-negative | Reference |  |  |
| **SMCQ3** |  |  |  |
| Model 1 ^a, *^ |  |  |  |
| SMC-positive | -0.203 | 0.120 |  |
| SMC-negative | Reference |  |  |
| Model 2 ^b, *^ |  |  |  |
| SMC-positive | -0.212 | 0.094 |  |
| SMC-negative | Reference |  |  |
| Model 3 ^c, *^ |  |  |  |
| SMC-positive | -0.207 | 0.105 |  |
| SMC-negative | Reference |  |  |
| **SMCQ4** |  |  |  |
| Model 1 ^a, *^ |  |  |  |
| SMC-positive | -0.264 | 0.025 |  |
| SMC-negative | Reference |  |  |
| Model 2 ^b, *^ |  |  |  |
| SMC-positive | -0.213 | 0.064 |  |
| SMC-negative | Reference |  |  |
| Model 3 ^c, *^ |  |  |  |
| SMC-positive | -0.198 | 0.115 |  |
| SMC-negative | Reference |  |  |

SMC=subjective memory complaint, Aβ=beta-amyloid, MCI=mild cognitive impairment, TS = total score of the consortium to establish a registry for Alzheimer’s disease neuropsychological battery, WLR= word list recall, API = amyloid prediction index.

^a^ Adjusted for age, sex, education, and geriatric depression scale status.

^b^ Adjusted for covariates in Model 1 plus, apolipoprotein ε4, hypertension, and diabetes mellitus.

^c^ Adjusted for covariates in Model 2 plus, body mass index status, alcohol intake, smoking, serum fasting glucose.

^*^Adjusted for covariates in Model plus API.
